# A Rare and Unusual Case of Burkitt's Lymphoma Presenting with a Prostate Mass in a 12-Year-Old Boy

**DOI:** 10.1155/2014/106176

**Published:** 2014-05-13

**Authors:** N. Sinclair, P. Babyn, M. Kinloch, R. Sinha

**Affiliations:** ^1^Department of Medical Imaging, Royal University Hospital, University of Saskatchewan, Room 1566, 103 Hospital Drive, Saskatoon, SK, Canada S7N 0W8; ^2^Department of Medical Imaging, University of Saskatchewan and Saskatoon Health Region, Saskatoon, SK, Canada S7N 0W8; ^3^Department of Pathology, University of Saskatchewan, Saskatoon, SK, Canada S7N 0W8; ^4^Department of Pediatrics, University of Saskatchewan and Saskatoon Health Region, Saskatoon, SK, Canada S7N 0W8

## Abstract

Burkitt's lymphoma is the most frequent subtype of non-Hodgkin's lymphoma in childhood. Radiographic findings are protean and can often overlap with other neoplastic and nonneoplastic processes. We present an unusual case of Burkitt's lymphoma in a 12-year-old boy presenting with a one-week history of urinary retention, dysuria, and “tailbone pain,” as well as a 4-week history of jaw pain, initially treated as a dental abscess. On dental radiography, the patient was found to have resorption of alveolar bone adjacent to the lower first molars bilaterally, in keeping with “floating teeth,” classically associated with Langerhans cell histiocytosis. Additionally, a large, eccentric, prostatic mass was noted, prompting the inclusion of rhabdomyosarcoma on the differential diagnosis, with subsequent definitive diagnosis of Burkitt's lymphoma on tissue and bone marrow biopsy. This case highlights the imaging overlap of these childhood neoplasms with an unusual lymphomatous prostate mass. It is important that the radiologists and pediatricians be aware of this potential overlap and the unusual presentation of Burkitt's lymphoma.

## 1. Introduction

Burkitt's lymphoma was first described in 1958, by the surgeon Denis Burkitt, who while working in Uganda, noted children with rapidly enlarging tumors of the jaw [1, 2]. The World Health Organization characterizes Burkitt's lymphoma into 3 types: endemic, sporadic, and immunodeficiency-associated [1]. Endemic Burkitt's lymphoma is associated with Epstein-Barr virus (EBV) in 95% of cases and is most commonly found in equatorial Africa and Papua New Guinea [1]. The sporadic (or American) type is associated with EBV only 15% of the time, while the immunodeficiency-associated type is seen in patients with HIV, allograft recipients, and those with congenital immunodeficiency [1].

Burkitt's lymphoma is the most frequent subtype of non-Hodgkin's lymphoma in childhood, with the jaw and abdomen, specifically terminal ileum, being the most common sites; it grows rapidly, with a doubling time of 24 hours [1]. Jaw involvement is common in the endemic type of Burkitt's lymphoma but far less common in the sporadic type [3].

## 2. Case Report

A 12-year-old Caucasian boy presented to the hospital with a four-week history of jaw pain, resulting in difficulty in eating. Upon presentation, he had developed gingivitis and bleeding gums. One week prior to admission, he developed pain in the tailbone area and noted difficulty in urinating with retention symptoms and periodic dysuria. In the week prior to admission, he had been seen at his home hospital emergency room and was started on antibiotics for a presumed dental infection. His family reported an approximate 15-pound weight loss in the month prior to admission. He denied fever but had mild night sweats. He reported low energy, for which he missed a week of school. He had been otherwise healthy. He takes no medications and reports no allergies and all immunizations were up to date. His past medical history and family history were noncontributory.

Head and neck exam revealed symmetric swelling around his lower incisors with upwards displacement of the teeth by almost 1.0 cm, both teeth were loose. There was no purulent discharge. There was swelling of the face and lower jaw area, without palpable lymphadenopathy. Note was made of hepatosplenomegaly, as well as bruises on his knees, shin, forearm, and elbow, felt to be from baseball. The physical exam was otherwise normal.

Admission blood work revealed platelets of 33 × 10^9^ L, WBC 15.9 × 10^9^ L, and hemoglobin of 141 g/L. Blood smear revealed a left shift with a few circulating blasts and abnormal cells. The liver enzymes were slightly elevated, as was the creatinine at 87 *μ*mol/L. The lactate dehydrogenase was markedly elevated at >2500 U/L, as was the uric acid at 1025 *μ*mol/L.

**Figure 1:**
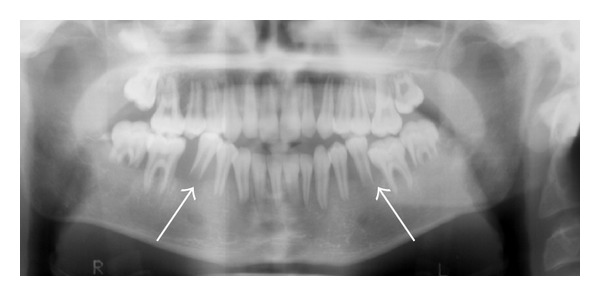
Panorex demonstrating resorption of alveolar bone of the lower first molars bilaterally with superior displacement of the teeth giving a “floating teeth” appearance.

Plain film imaging of the jaw performed at an outside institution revealed loss of alveolar bone adjacent to the roots of the lower first molars bilaterally with erosion of the distal roots at these levels, in keeping with “floating teeth” (Figure 1).

**Figure 2:**
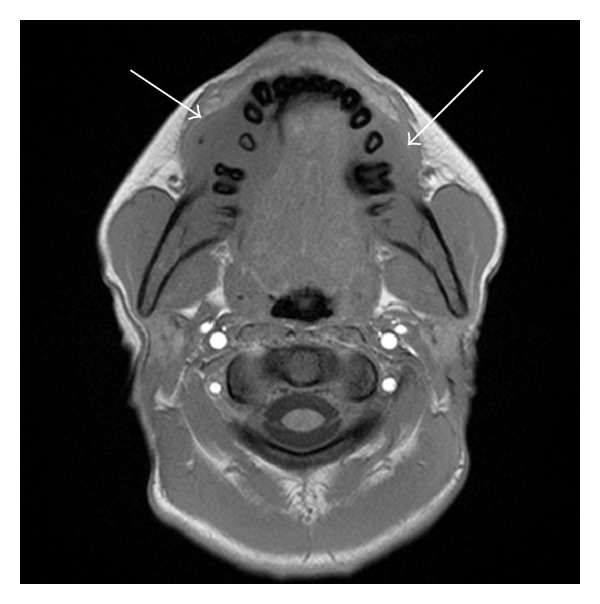
Axial T1 and T1 FS after contrast, demonstrating isointense, enhancing mandibular masses, cortical erosion, and adjacent soft tissue mass.

**Figure 3:**
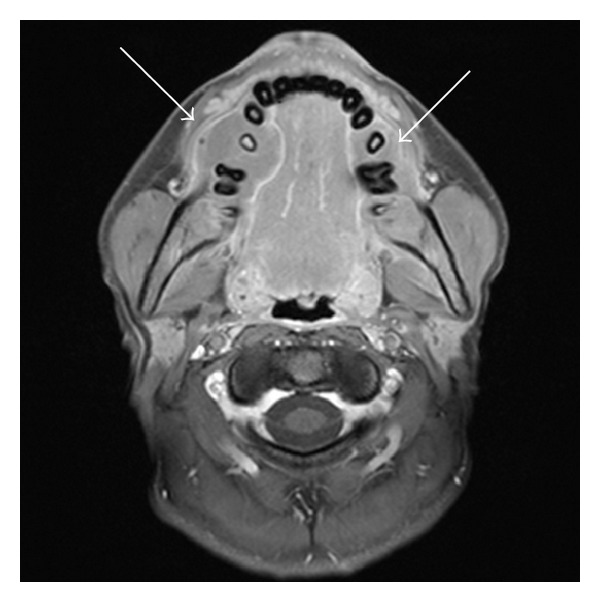
Axial T1 and T1 FS after contrast, demonstrating isointense, enhancing mandibular masses, cortical erosion, and adjacent soft tissue mass.

An MRI of the brain, face/palate, and pelvis was performed, which revealed multiple lesions within the mandible and maxilla. These lesions were slightly T2 hyperintense and T1 isointense to muscle with homogenous enhancement. There was involvement of the body of the mandible bilaterally, extending superficially and deeply to the mandibular margins with cortical erosion. Maxillary lesions were also present, extending to the anterior margins of the maxilla, again with cortical erosion (Figures 2 and 3). The brain and pituitary gland were normal.

**Figure 4:**
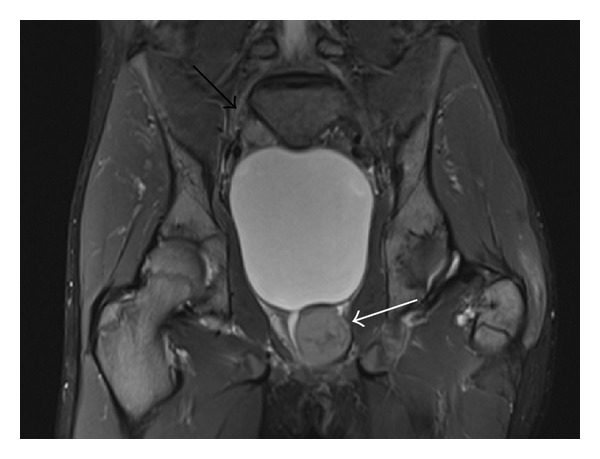
Coronal STIR shows 3 × 4 cm mildly hyperintense mass with central linear hypointensity within left lobe of prostate causing deviation of ureter and bladder outlet obstruction (white arrow). A 2 cm mass superior/posterior to the urinary bladder demonstrates similar signal characteristics (black arrow). Diffusely abnormal increased bone marrow signal is also noted.

**Figure 5:**
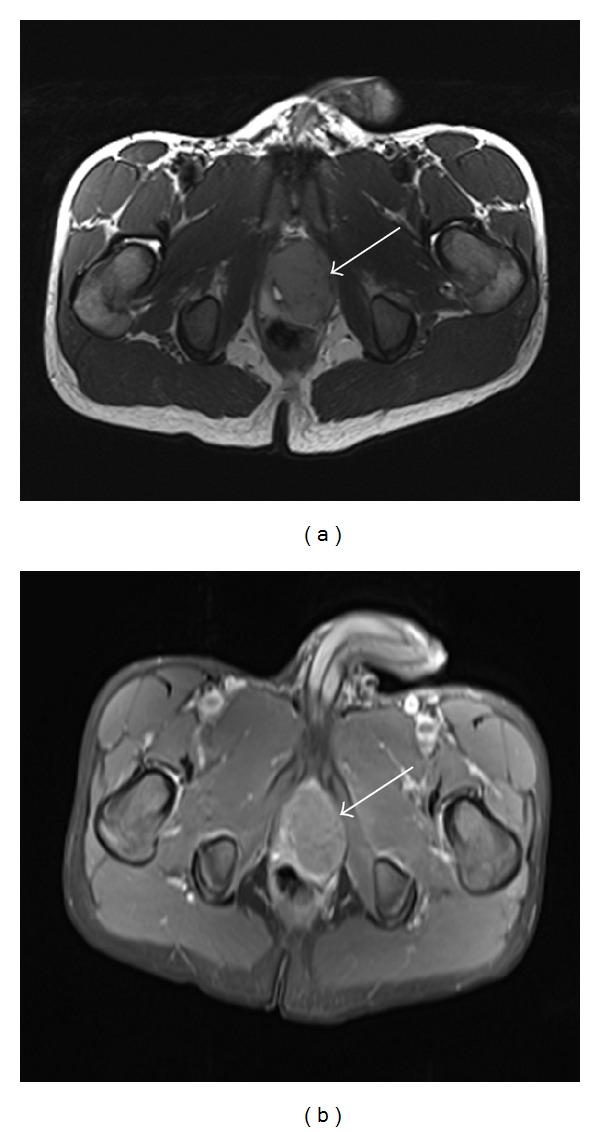
Axial T2 and T1 FS postcontrast images show mildly T2 hyperintense, well- circumscribed, enhancing lesion arising from left lobe of prostate.

**Figure 6:**
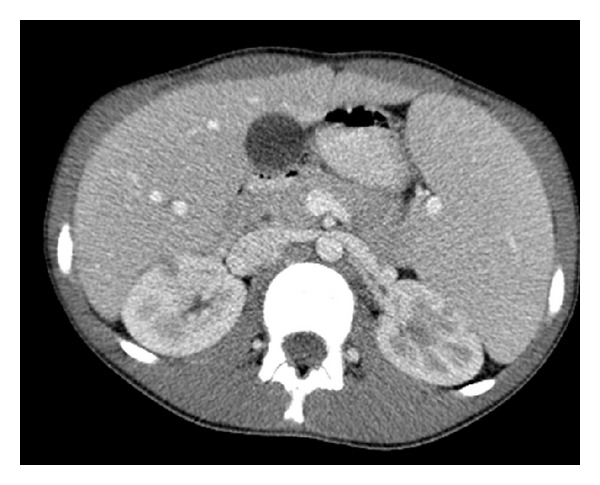
Axial contrast enhanced CT shows multiple, small, bilateral hypodense kidney lesions in keeping with lymphomatous involvement.

Within the pelvis, there was a well-circumscribed, periurethral mass within the left lobe of the prostate gland (3 × 4 × 4 cm) demonstrating slight T2 hyperintensity to muscle with irregular, linear central hypointense regions, and faint enhancement on the postcontrast T1 FS images. As well, a 2 cm mass at the superior/posterior aspect of the bladder on the right (Figures 4, 5(a), and 5(b)) was identified, demonstrating similar signal characteristics. Hepatosplenomegaly was also noted. A contrast enhanced CT of the chest, abdomen, and pelvis demonstrated multiple, bilateral subcentimeter hypodense lesions involving both kidneys (Figure 6). On CT, the prostate demonstrated asymmetric enlargement with urethral deviation to the right. An enlarged left internal mammary lymph node was also noted. A bone scan was performed and demonstrated normal radiotracer uptake.

**Figure 7:**
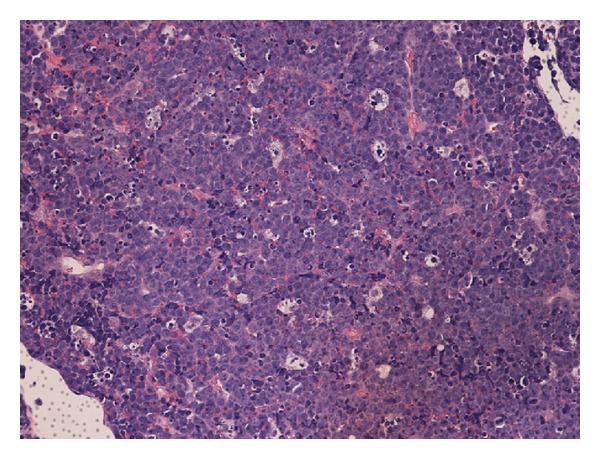
20× power H&E stain jaw biopsy. Diffuse infiltrate of medium to large lymphoid cells that are monomorphic against a heavy background of scattered macrophages with a “starry sky” pattern, in keeping with Burkitt's lymphoma.

A right mandibular biopsy showed diffuse infiltrate of medium to large lymphoid cells that were monomorphic against a heavy background of scattered macrophages with a “starry sky” pattern (Figure 7). Bone marrow biopsy from the right iliac crest showed more than 95% pattern of infiltration with Burkitt lymphoma, which was confirmed with immunophenotyping of the neoplastic cells in the marrow. Cerebrospinal fluid showed clusters of cells with degeneration that were similar to the known neoplastic cells.

The patient was diagnosed with stage IV Burkitt's lymphoma and COP reduction chemotherapy was initiated immediately. The patient experienced a dramatic reduction in his tumor burden, with follow-up imaging of the prostate revealing near complete resolution of the mass. Unfortunately, the patient relapsed with Burkitt's Leukemia approximately 6 months after initial treatment was started, with 99% blast involvement of his bone marrow. During ongoing therapy, the patient experienced sepsis in the context of profound pancytopenia, acute kidney injury requiring continuous renal replacement therapy, enterococcus pneumonia requiring intubation, and a large pericardial effusion. On day 15 after cycle number 2 of his chemotherapeutic regime for Burkitt's leukemia, he developed severe lactic acidosis, respiratory failure, and severe, profound bradycardia that could not be reversed. He died the following morning, seven and a half months from his initial diagnosis.

## 3. Discussion

Though the most common type of non-Hodgkin's lymphoma in children, prostate involvement of Burkitt's lymphoma is uncommon and accounts for <0.1% of genitourinary involvement [4]. In a multi-institutional study of 62 cases of malignant lymphoma involving the prostate, only one case was found to be Burkitt's lymphoma; this happened to be in the single child [5]. In this case series, there was a 5-year-old boy with secondary involvement of the prostate by Burkitt's lymphoma, who died 1 week after diagnosis. The imaging findings in this case were not described.

The terminal ileum is the most common location of Burkitt's lymphoma in children [1]. Though abdominal and pelvic involvement are common, prostatic involvement of Burkitt's lymphoma, specifically in children, has not been previously described in the imaging literature. In their report of 62 cases of malignant lymphoma involving the prostate, predominantly in adults, Bostwick et al. found that secondary involvement of the prostate was more common than primary involvement (65% versus 35%), and that lymphoma specific survival was 64% at 1 year and 50% at 2 years [5].

Specific imaging of the prostate gland is rarely warranted in children but is included during workup of symptoms related to the lower genitourinary tract, including urinary retention, hematuria, dysuria, and incontinence, or during investigations for suspected congenital anomalies [6]. In children presenting for workup of a prostatic mass, rhabdomyosarcoma would be high on the differential diagnosis, as it is the most common tumor of the lower genitourinary tract in children and often involves the prostate gland [6, 7]. Other pediatric prostatic tumors are extremely rare [5, 8, 9]. These children often present with symptoms of urinary and fecal retention. Rhabdomyosarcomas originating in the prostate carry significantly worse prognosis than do tumors that involve bladder only [8]. Bladder wall invasion may be detected on MR imaging, with T2-weighted images demonstrating higher signal intensity tumor extending into the lower-signal intensity wall. Perivesical and perirectal fat invasion can be demonstrated on T1-weighted images [6].

Leukemic infiltration has a similar MR appearance to lymphoma, with hypovascularity and only mild contrast enhancement [6]. A case of myeloid sarcoma of the prostate in a child with acute myelogenous leukemia has been reported [9]. MR imaging was not performed in this case, with sonography showing a hypoechoic mass involving the left bladder wall displacing the rectum posteriorly. This lesion was irregular and heterogeneously enhancing on contrast enhanced CT [9].

Prostatic carcinoma and carcinoid have also been reported in the pediatric population [10, 11]. A case of primary carcinoid of the prostate in a 7-year-old boy with multiple endocrine neoplasia IIb has been reported, described as a T2 hyperintense mass without extension beyond the prostate [11]. Inflammatory tumor of the prostate in a child has been reported as a cystic lobular and centrally necrotic midline tumor that nearly completely resolved with antibiotic therapy [12]. Chronic prostatitis is rare and may be secondary to abnormal voiding conditions [6].

As previously mentioned, jaw involvement with Burkitt's lymphoma is more commonly seen with the endemic form though a case describing “floating teeth” at presentation in sporadic Burkitt's lymphoma in a 66-year-old male has been published [13]. Historically, “floating teeth” in pediatric patients have been thought to be almost pathognomonic of Langerhans cell histiocytosis, though this finding may also reflect any destructive process in the mandible, including infectious, hematologic, metabolic, or neoplastic etiologies [14]. In our case, the presence of floating teeth on plain radiography prompted not only the MR imaging of the jaw but also the pituitary, which can also be involved in Langerhans cell histiocytosis and is often associated with diabetes insipidus [15].

Though a definitive diagnosis was swiftly made on tissue and bone marrow biopsy, this case highlights the imaging overlap of these childhood neoplasms. It is important that the radiologist and pediatrician be aware of these similarities and that not all pediatric prostatic masses reflect rhabdomyosarcoma. Definitive diagnosis requires histologic examination in all cases.

## References

[1] Biko DM, Anupindi SA, Hernandez A, Kersun L, Bellah R (2009). Childhood Burkitt lymphoma: abdominal and pelvic imaging findings. *American Journal of Roentgenology*.

[2] Ferry JA (2006). Burkitt’s lymphoma: clinicopathologic features and differential diagnosis. *Oncologist*.

[3] Toma P, Granata C, Rossi A, Garaventa A (2007). Multimodality imaging of Hodgkin disease and non-Hodgkin lymphomas in children. *Radiographics*.

[4] Pereira Arias JG, Prieto Ugidos N, Larrinaga Simon J (1997). Primary prostatic infiltration by Burkitt’s lymphoma. *Archivos Españoles de Urología*.

[5] Bostwick DG, Iczkowski KA, Amin MB, Discigil G, Osborne B (1998). Malignant lymphoma involving the prostate: report of 62 cases. *Cancer*.

[6] Mong A, Bellah R (2006). Imaging the pediatric prostate. *Radiologic Clinics of North America*.

[7] Agrons GA, Wagner BJ, Lonergan GJ, Dickey GE, Kaufman MS (1997). From the archives of the AFIP genitourinary rhabdomyosarcoma in children: radiologic-pathologic correlation. *RadioGraphics*.

[8] McCarville MB, Spunt SL, Pappo AS (2001). Rhabdomyosarcoma in pediatric patients: the good, the bad and the unusual. *American Journal of Roentgenology*.

[9] Joshi A, Patel A, Patel K (2005). Myeloid sarcoma of prostate with urinary obstruction: an unusual presentation of acute myelogenous leukemia in a child. *Indian Journal of Medical and Paediatric Oncology*.

[10] Shimada H, Misugi K, Sasaki Y (1980). Carcinoma of the prostate in childhood and adolescence: report of a case and review of the literature. *Cancer*.

[11] Whelan T, Gatfield CT, Robertson S, Carpenter B, Schillinger JF (1995). Primary carcinoid of the prostate in conjunction with multiple endocrine neoplasia IIb in a child. *Journal of Urology*.

[12] Hacker H-W, Winiker H, Caduff J, Schwoebel M-G (2009). Inflammatory tumour of the prostate in a 4-year-old boy. *Journal of Pediatric Urology*.

[13] Murrin RJA, Mahendra P (2004). “Floating” teeth at presentation in sporadic Burkitt’s lymphoma. *British Journal of Haematology*.

[14] Keusch KD, Poole CA, King DR (1966). The significance of “floating teeth” in children. *Radiology*.

[15] Meyer JS, Harty MP, Mahboubi S (1995). Langerhans cell histiocytosis: presentation and evolution of radiologic findings with clinical correlation. *Radiographics*.

